# Lexical Profile of Newspapers Revisited: A Corpus-Based Analysis

**DOI:** 10.3389/fpsyg.2022.800983

**Published:** 2022-02-24

**Authors:** Hung Tan Ha

**Affiliations:** School of Foreign Languages, University of Economics Ho Chi Minh City (UEH), Ho Chi Minh City, Vietnam

**Keywords:** lexical coverage, vocabulary profile, BNC, COCA, News on the Web

## Abstract

The present study analyzed the vocabulary profile of the News on the Web (NOW) corpus, which contained 12 billion words from online newspapers and magazines in 20 countries to determine the vocabulary knowledge needed to reasonably understand online newspaper and magazine articles. The results showed that, in general, knowledge of the most frequent 4,000 word families in the British National Corpus/Corpus of Contemporary American English (BNC/COCA) wordlist plus proper nouns, marginal words, transparent compounds and acronyms was necessary to gain 95% coverage for the NOW corpus. However, when it came to the 98% coverage, online newspaper and magazine articles from different countries had relatively distinct lexical demands. In-depth analyses were carried out and the findings offered comprehensive insights into the issue. Implications for teaching and learning were also provided.

## Introduction

Newspapers have been a crucial of part people’s lives, with 52–85% of the people in different countries reading news more than once a day (Cabrera, 2020). Together with the development of technology, most aspects of our lives have been digitized, and the way we receive our daily news is certainly no exception. Researches have shown that people are giving up traditional, paper-based news and giving their favor to its digital, online counterparts (Cabrera, 2020; Pew Research Center, 2021a,b). In a recent research conducted on American news habit by Shearer (2021), 86% of United States adults got their news from a digital device (e.g., smartphone, computer, or tablet). These figures were significantly higher than television (68%), radio (50%) and print publications (32%) (Shearer, 2021). The main reason for this shift could be due to convenience. Compared to print newspapers or magazines, online publications are more easily accessible, could be read from anywhere and are supported by state-of-the-art technology which offers better reading experience. On top of that, most, if not all, online newspapers are eco- and reader-friendly, that is, they are free to read and do not harm any tree.

Due to the popularity of online newspapers, the ability to read and comprehend this type of publication has been viewed as a critical goal for second language learning, and the use of newspapers has always been emphasized in language teaching (Nation, 2013; Nation and Macalister, 2021). In fact, many language proficiency tests, like IELTS, have long incorporated newspaper and magazine articles in their reading component (Moore et al., 2011, 2015). As a result, it is crucial for English teachers and learners to be informed of the amount of vocabulary necessary to comprehend an online newspaper article, such a knowledge would strongly inform various decisions in goal setting, lesson planning and course book design. The aim of this manuscript is to offer an answer to such question, that is, to determine the number of words required to understand online newspaper or magazine articles.

## Literature Review

### Vocabulary Demand and Comprehension

Vocabulary is the most important aspect in language and plays a fundamental role in most if not all language abilities or skills (Laufer and Ravenhorst-Kalovski, 2010; Schmitt et al., 2011; van Zeeland and Schmitt, 2013; Cheng and Matthews, 2018; Lange and Matthews, 2020; Qian and Lin, 2020; Ha, 2021b). In fact, the lexical resource of learners has been proven to be of even greater importance to their comprehension compared to the knowledge of grammatical structures and subject matters (Lewis, 2002; Barcroft, 2007; Guo and Roehrig, 2011; Zhang, 2012; Zhang and Koda, 2013). As Wilkins (1972) pressed, “[…] while without grammar very little can be conveyed, without vocabulary nothing can be conveyed” (pp. 111, 112).

The term “lexical demand” is now quite familiar in the field of applied linguistics and vocabulary these days. The idea behind the terminology is that a reader need to know a certain proportion of words in a text in order to reasonably comprehend it (Nation, 2013; Webb and Nation, 2013). In general, it has been widely accepted that readers or listeners of different text genres need to be familiar with at least 95% and preferably 98% of the running words in a text to gain adequate comprehension (Laufer and Ravenhorst-Kalovski, 2010; Schmitt et al., 2011; Laufer, 2013; van Zeeland and Schmitt, 2013). Despite a small gap of only 3% coverage, the difference between the two thresholds could be far more significant than some people may think. Simply speaking, with a 95% coverage, readers would encounter an unfamiliar word in every twenty words, but that ratio would be down to 1/50 if they were to know 98% of what they were reading (Hu and Nation, 2000). In other words, the 98% coverage would reduce more than half of the unknown words in the text which would be encountered if readers only had 95% coverage. In support of that claim, studies on the lexical demands of various text genres pointed out that learners who have vocabulary knowledge at the 95% threshold would have to double or even triple their lexical resources if they wished to gain 98% coverage of the same text genres (Nation, 2006; Coxhead and Walls, 2012; Webb and Macalister, 2013; Dang and Webb, 2014; Nurmukhamedov, 2017; Tegge, 2017). Hu and Nation (2000) also stated that 98% was the desirable threshold for adequate comprehension while 95% was only the acceptable threshold for minimal comprehension in which some may gain adequate comprehension but most may not.

Research have also shown a close relationship between lexical coverage and language teaching, especially when selecting materials for reading-related activities. According to Nation’s (2007) principles of the four strands, for language-focused or form-focused instructions, it is suggested that learners should know no less than 85% of the words in their reading texts (Schmitt et al., 2011; Stoeckel et al., 2020). If the purpose involved supported reading comprehension, a 95% coverage would be demanded (Laufer, 1989; Schmitt et al., 2011). For meaning-focused or extensive reading, learners would be required to be familiar with 98% of the tokens in their reading materials (Nation, 2007; Webb and Nation, 2017). And for fluency development, a coverage threshold of 100% would be necessary (Nation, 2007).

### Word-Frequency Lists

One of the reasons why findings of lexical profiling researches are of so much interest to linguists is because they are based on word-frequency lists. These lists classify English words into several 1,000-word levels according to how frequent they appear in authentic texts, which offers teachers and learners of English a clear and fast route to their learning goal (Nation, 2013). The British National Corpus (BNC) lists that contain fourteen 1,000-word levels (Nation, 2006) and the British National Corpus/Corpus of Contemporary American English (BNC/COCA) lists (Nation, 2017) that consist of twenty-five 1,000-word levels are typical examples of these wordlists.

Most of these wordlists were built on a word counting unit called “word family” which refers to a headword and all of its inflectional and derivational forms through a level 6 affix criteria (also known as WF6) (Bauer and Nation, 1993; Nation, 2020). The rationale for using WF6 was based on the assumption of learning burden, that was, when a learner knew a family member, he or she could understand or recognize the rest of the family with little or zero effort (Nation, 2013; Laufer and Cobb, 2020; Laufer, 2021; Laufer et al., 2021). It is worth noting that the WF6 have served as a basis for most aspects of vocabulary researches including assessment (McLean and Kramer, 2015; McLean et al., 2015; Webb et al., 2017; Ha, 2021a) and other psycholinguistic areas (Laufer and Ravenhorst-Kalovski, 2010; Lange and Matthews, 2020; Qian and Lin, 2020; Ha, 2021b).

### Lexical Demands of Written and Spoken Texts

Over decades, researchers in the field of vocabulary studies have documented a sound lexical profile of various spoken text genres. For example, Webb and Rodgers (2009a,b) told us that learners would need to know the most frequent 3,000 and 7,000 word families in the BNC list to understand 95 and 98% of the words in movies and TV programs, respectively. These figures aligned really well with what we would need to comprehend daily conversations (Nation, 2006). It seemed that the language people used when they were in the stage did not differ much from what they used in their everyday talking. Songs, Soap opera, sitcom and podcast were relatively less demanding as they only required approximately 2,000–3,000 word families for 95% coverage, and 5,000–7,000 for 98% coverage (Al-Surmi, 2014; Tegge, 2017; Nurmukhamedov and Sharakhimov, 2021). When we decided to take things a little bit more serious and look at spoken discourses in academic contexts, some scholars would be happy to give us the answers. For instance, to understand 95 and 98% of the words in academic lectures and seminars, audience would need to have a lexical resource equivalent to 4,000 and 8,000 word families in the BNC word list, correspondingly (Dang and Webb, 2014). It was interesting to see that TED talks would share the same lexical demands (Coxhead and Walls, 2012; Nurmukhamedov, 2017).

Compared to spoken discourses, the lexical profile of written texts received relatively less attention. In Hsu (2011) pointed out that 5,000 and 8,000 most frequent word families in Nation’s (2006) BNC word list would account for 95 and 98% of the words in business textbooks and business research articles. Seven years later, Hsu (2018) examined the lexical coverage of English-written Chinese medicine textbooks and found that 10,000 most frequent word families in Nation’s (2017) BNC/COCA word list would provide 98% coverage for the corpus. In 2013, Webb and Macalister examined the difference in lexical demands between written literatures for native English speakers and learners of English as a second language. Their results showed that, at 95% coverage threshold, only 3,000 and 2,000 most frequent word families in the BNC list was required for L1 and L2 literature, correspondingly, signaling a small difference of only 1,000 word families. However, at the 98% threshold, while L2 literature only required a vocabulary knowledge at the 3,000 level, written texts for L1 learners needed the lexical knowledge at 10,000 level, which was more than triple. In attempts to provide updates on the vocabulary profile of textbooks for English as a foreign language (EFL) learners, researchers have found that the knowledge of 3,000–4,000 most frequent word families in Nation’s (2017) BNC/COCA was sufficient to provide 95% coverage, and for learners to understand 98% of the words in those books, a word knowledge at 5,000–6,000 levels were required (Yang and Coxhead, 2020; Rahmat and Coxhead, 2021).

The most influential manuscript that investigated the lexical demand of written English was undoubtedly Nation’s (2006) study. In his study, Nation (2006) found that learners would need about 4,000 most frequent word families in the BNC list plus proper nouns to reach 95% coverage in newspapers and novels, and approximately 8,000–9,000 word families plus proper nouns to gain 98% coverage. Despite the impact given by his study, those figures demand to be revisited for two reasons. First, this research was carried out approximately 15 years ago using a relatively small corpus (only 440,000 words), and therefore, those findings “now need to be checked with larger, more comprehensive corpora” (Schmitt et al., 2017, p. 217). The second reason lies with the methodology Nation (2006) used for indicating vocabulary size. In his study, Nation (2006) utilized the BNC wordlist based entirely on British English which “may be due for updating and revision” (Schmitt et al., 2017, p. 218). Fortunately, Nation made significant effort in improving his wordlists which eventually resulted in the introduction of BNC/COCA in 2012, which was updated in 2017 (Nation, 2017). The BNC/COCA is a very powerful wordlist that covers both British and American Englishes and are proven to outperform other wordlists (Dang and Webb, 2016; Dang et al., 2020). As Schmitt et al. (2017) wrote, “Assuming the new combined BNC-COCA lists are a better indication of word frequency, then everything that has been done using the original BNC-based lists is ripe for replication using these new lists” (p. 218).

## The Present Study

The present study sets out to revisit Nation’s (2006) figures following the two major suggestions put forward by Schmitt et al. (2017): increasing sample size and employing up-to-date research methodology.

To re-examine the lexical profile of newspapers from the perspective of a larger sample size, the present study analyzed Davies’s (2016/2021) News on the Web (NOW) corpus, the largest corpus of English newspapers available. Besides the ultra-large sample size, the current study also employed the most comprehensive and up-to-date BNC/COCA wordlist (Nation, 2017). The word list contains twenty-five 1,000-word levels which reflects current English. In addition, the BNC/COCA is accompanied by four supplementary lists of proper nouns (*Aaron, Greece, Grecian, Greenberry*, and *Waterloo…*), marginal words (*hm, huh, er, ah*, and *phew…*), transparent compounds (*aftershock, afterword, airbag*, and *powerboat…*) and acronyms (*PHD, UFO*, and *UDA…*) (Nation, 2020), which allow more detailed analyses compared to the BNC list which is only accompanied by two supplementary lists of proper nouns and marginal words.

Moreover, the use of the BNC/COCA lists in lexical profiling research also contributes to the methodological shift in the field of vocabulary studies. As the most widely used vocabulary tests have long utilized the BNC/COCA lists as the source for their test items (McLean and Kramer, 2015; McLean et al., 2015; Webb et al., 2017), it would not take long for researches on most aspects of vocabulary knowledge and lexical development to follow. Therefore, it would be methodologically inconsistent to relate the lexical profile of a text based on the BNC lists to a study that reflected learners’ vocabulary knowledge of the BNC/COCA lists.

The study also responds to Webb’s (2021) call for more attention to the variation in lexical demands. In a recent manuscript, Webb (2021) expressed his concern that lexical profiling researches only “reflect the mean number of word families needed to reach a certain lexical coverage figure” and often ignore the fact that “each corpus is made up of a large number of texts, and there is likely to be a great deal of variation in the vocabulary of each text” (p. 286). It is true that we should not assume the same coverage to be reliably applied on different texts just because they belong to the same text genre, especially for newspapers. Researches have shown that the use of grammar and vocabulary could greatly vary among regions and generations (Chambers, 2000; Davies and Fuchs, 2015; Davies, 2021; Wan and Cowie, 2021). Therefore, it would be reasonable to hypothesize that the lexical demands of newspaper and magazine articles from different countries and periods of time bear certain degrees of distinction. The NOW corpus comprises data from online newspapers and magazines collected from twenty countries over a period of 11 years. The analysis of such corpus not only provides reliable figures on the lexical demand of newspapers, but also offers deep insights into the variation of the vocabulary knowledge required to comprehend English newspapers written in different countries and years.

In particular, the study seeks to answer the following questions:

(1) *How many words do English learners need to gain 95 and 98% coverage of online newspapers?*
(2) *Does the lexical profile of online newspapers and magazines vary over time and across countries?*

## Methodology

### Data Collection

The present study analyzed data from The NOW corpus (Davies, 2016/2021), the corpus is available for purchase on Mark Davies’s website.^1^ The NOW corpus contains data from articles on web-based newspapers and magazines collected from twenty different countries. The corpus has been continuously updated from 2010 to the present time and grows approximately 200 million words per month, which is equivalent to three or four hundred thousand articles. At the time of data collection, May 2021, the NOW corpus contains approximately 12.5 billion words of data. The full-text data of the corpus was purchased by the researcher and license for academic use was appropriately obtained.

### Data Preparation

A preliminary analysis was carried out for the NOW corpus by a lexical profiler software (Heatley et al., 2002). After that, two major adjustments were made to the corpus. Firstly, words that were falsely classified as “Not in the lists” due to spelling errors or typos were corrected and returned to their frequency levels. Secondly, since the lexical profiler software cannot read hyphenated words (e.g., *full-time, second-hand, money-driven, customer-focus*, etc.), hyphens in the texts were replaced by spaces so that the words that made up hyphenated items could be classified in accordance with their frequency (e.g., *second, hand, money, customer, focus*, etc.). These processes were done using the mass replace (or Ctrl + Shift + F) function of Notepad++.

### Data Analysis

The RANGE program (Heatley et al., 2002) was used for data analysis. RANGE classifies all the words in a text to their frequency levels according and the number of times they were used. The “frequency” that RANGE would base its lexical analysis on depends on the wordlist they are being used with. In other words, RANGE allows us to know exactly the number of words at each level in a wordlist, which would later facilitate various conclusions and predictions. Currently, there are three wordlists that can be used with RANGE: The General Service List/Academic Word List (GSL/AWL) which include 2,570 word families, the BNC wordlist consisted of fourteen 1,000-word levels plus two levels of proper nouns and marginal words, and the BNC/COCA wordlist which contains twenty-five lists of word families from the 1,000 to 25,000 levels plus four additional lists of proper nouns, marginal words, transparent compounds and acronyms.

The current study utilizes the BNC/COCA word list (Nation, 2017). RANGE is available at: https://www.wgtn.ac.nz/lals/resources/paul-nations-resources/vocabulary-analysis-programs. The RANGE program automatically read and recognized contractions (can’t, don’t…) and connected speech (wanna, gonna, and kinda…). For instance, RANGE counted the word *don’t* as two separated words of *do* and *not* and *wanna* as a family member of *want.*

## Results

The second and third columns of Table 1 present the coverage of each word level for the NOW corpus. The most frequent 1,000 word families in the BNC/COCA wordlist accounted for the greatest proportion of tokens, 72.48%. The coverage then dropped significantly to 10.27% at the second 1,000-word level. After the 2,000 word families level, the number of tokens as well as its coverage gradually decreased as the word frequency went down. Lower-frequency levels from the 5,000 level onward only accounted for less than 1% of the running words in the corpus, which generally highlighted the importance of high-frequency words to reading comprehension.

**TABLE 1:** The proportions of tokens each word level and the cumulative coverage with and without proper nouns, marginal words, transparent compounds, and acronyms for the News on the Web (NOW) corpus.

Word list	Tokens	Coverage at each level (%)	Cumulative coverage without PN, MW, TC, and acronym (%)	Cumulative coverage with PN, MW, TC, and acronym (%)
1,000	8,616,263,239	72.48	72.48	77.87
2,000	1,221,457,304	10.27	82.75	88.15
3,000	739,364,222	6.22	88.97	94.37
4,000	206,631,853	1.74	90.71	96.11^a^
5,000	115,906,531	0.98	91.69	97.08
6,000	79,362,050	0.67	92.35	97.75
7,000	52,221,650	0.44	92.79	98.19^b^
8,000	46,147,921	0.39	93.18	98.58
9,000	29,320,140	0.25	93.43	98.82
10,000	19,053,027	0.16	93.59	98.98
11,000	16,225,949	0.14	93.73	99.12
12,000	12,531,144	0.11	93.83	99.23
13,000	8,788,706	0.07	93.91	99.30
14,000	8,608,025	0.07	93.98	99.37
15,000	4,628,179	0.04	94.02	99.41
16,000	4,451,764	0.04	94.05	99.45
17,000	3,129,096	0.03	94.08	99.47
18,000	3,778,722	0.03	94.11	99.51
19,000	2,329,532	0.02	94.13	99.53
20,000	2,432,275	0.02	94.15	99.55
21,000	1,674,960	0.01	94.17	99.56
22,000	1,248,712	0.01	94.18	99.57
23,000	3,314,878	0.03	94.20	99.60
24,000	1,613,907	0.01	94.22	99.61
25,000	1,660,694	0.01	94.23	99.63
Proper nouns	465,033,862	3.91	3.91	–
Marginal words	59,592,391	0.50	0.50	–
Transparent compounds	69,863,500	0.59	0.59	–
Acronyms	46,806,237	0.39	0.39	–
Not in the lists	44,385,236	0.37	0.37	100
Total	11,887,825,705			

*^a^Reaching 95% coverage; ^b^Reaching 95% coverage.*

Another worth noting detail was the proportion of proper nouns, marginal words, transparent compounds and acronyms in the corpus. Proper nouns alone were found to make up of 3.91% of the tokens in the NOW corpus. The combined coverage of PN, MW, TC, and acronyms made up of 5.39% of the running words, which was close to the coverage provided by the third most frequent 3,000 word families in the BNC/COCA word list. These figures demonstrate the relative importance of being able to recognize and understand proper nouns as well as marginal words, transparent compounds and acronym.

The last two columns of Table 1 show the vocabulary knowledge needed to reach 95 and 98% coverage of online newspapers. The results from the analyses displayed two hypothesized scenarios: one assumed that all proper nouns, marginal words, transparent compounds and acronyms were easily understood or recognized, and one supposed that they were not. Since more than 5% of the tokens accounted by the four supplementary lists, it was more than certain that understanding 95% of the running words in online newspapers with the sheer knowledge of the twenty-five thousand word families in Nation’s (2017) BNC/COCA lists was impossible. However, if proper nouns, marginal words, transparent compounds and acronyms were assumed to be known, then the knowledge of 4,000 and 7,000 most frequent word families were necessary to achieve 95 and 98% coverage, respectively.

However, when looking at the NOW corpus from another angle, we could easily realize that the corpus was made up of newspaper and magazine articles from twenty different countries over a period of 11 years. Therefore, it may not be appropriate to judge the corpus’s lexical demand by its twelve billion tokens in combination, and the lexical profile of the corpus deserves a deeper investigation into its variation. Supplementary Appendix offers data for such analysis.

**FIGURE 1:**
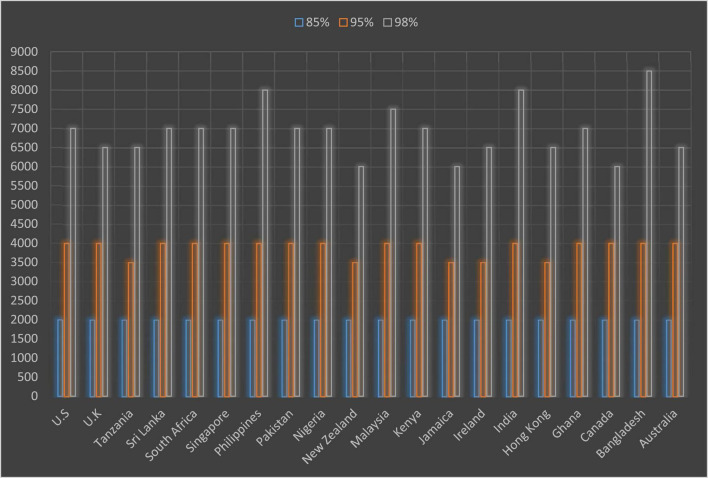
The amount of vocabulary needed to achieve 85, 95, and 98% coverage for online newspapers.

Supplementary Appendix provides data about the cumulative coverage of each sub-corpora including proper nouns, marginal words, transparent compounds and acronyms. Results from the analyses of the sub-corpora yielded interesting findings. Figure 1 is a graphic representation of Supplementary Appendix and offers visual support for the amount of words needed to achieve 85, 95, and 98% coverage for online newspapers in different countries. Due to space limitation, Figure 1 can only demonstrate a rough summary of the vocabulary demands of newspaper and magazine articles in different nations. Several “middle” numbers like 3,500 or 6,500 could be spotted in Figure 1, which did not necessarily mean that 3,500 or 6,500 word families were needed to understand 95 or 98% of the words in newspapers in certain countries. In fact, these “middle” figures signaled that a vocabulary knowledge of 3,000–4,000 or 6,000–7,000 word families was required for these coverage thresholds. This was due to the variation of vocabulary demands between different years or even between periods of time. Take Hong Kong as an example, although figures from 2012 to 2021 suggested that the 4,000 most frequent word families in the BNC/COCA lists were needed for 95% coverage, data from 2010 to 2011 showed us that it only took 3,000 word families to reach the same threshold.

It could be observed that 2,000 most frequent word families in the BNC/COCA wordlist covered 85–90% of the running words in online newspaper and magazine articles from all 20 countries. This highlighted the feasibility of using web-based newspapers as reading materials in English classes as well as the value of the BNC/COCA 2,000 to English learners. Although a one-size-fits-all threshold for 95 and 98% coverage could be said to be fictional, the data suggested that the vocabulary knowledge of the most frequent 3,000–4,000 word families in the BNC/COCA word list was necessary to gain 95% coverage. However, things became more complicated when it came to the 98% coverage. For some countries like Jamaica, New Zealand and Canada, the vocabulary knowledge at 6,000 level was enough to provide 98% coverage of web-based newspapers and magazines. Online newspaper and magazine articles from countries including Australia, Hong Kong, Ireland, Tanzania, and the United Kingdom seemed to be a little more demanding and required 6,000–7,000 for optimal comprehension. Online newspapers and magazines in the United States, South Africa, Singapore, Pakistan, Nigeria, Kenya, and Ghana needed the knowledge of the most frequent 7,000 word families to comfortably understand. For Malaysia and Sri Lanka, the required vocabulary knowledge for the 98% threshold was at 7,000–8,000 levels. Online newspapers and magazines written by publishers based in India and Philippines required readers to have a vocabulary knowledge at 8,000 level for optimal comprehension.

Bangladesh’ was the most unique country which required a range of vocabulary knowledge from 8,000 to 10,000 word families for 98% coverage. It was also worth noting that online newspapers and magazines in Bangladesh only needed a knowledge at 4,000 level for 95% coverage. The reason for the difference between the lexical demands for 95 and 98% coverage of Bangladesh’s newspapers might lie with the substantial proportion of words at the 13,000 level. Normally, lexical coverage at the 10,000 level and higher dropped below 0.2 or even 0.1 for most country sub-corpora in the NOW corpus. The same also went for the Bangladesh sub-corpus when the tenth, eleventh and twelfth 1,000-word level only represented the coverage of less than 0.2%. However, the coverage of the 13,000 level went up to 0.45–0.68% for the Bangladesh sub-corpora, which were relatively high for such a low-frequency word-level.

## Discussion

In answer to the first research questions, the vocabulary knowledge of the most frequent 4,000 families in the BNC/COCA list plus proper nouns, marginal words, transparent compounds and acronyms would reliably provide 95% coverage of the NOW corpus. This means that if 95% coverage was assumed to be sufficient for reasonable comprehension, then learning the most frequent 4,000 word families would be the ultimate learning goal for English learners in ESL and EFL contexts. As Laufer and Ravenhorst-Kalovski (2010) and Schmitt et al. (2011) suggested, 95% coverage of a text would result in acceptable comprehension, however, they also pointed out that the degree of comprehension at the 95% threshold is not really reliable and that 98% was truly the threshold for unsupported comprehension. If 98% were supposed to be the necessary threshold for text comprehension, then a one-size-fits-all answer would be nearly impossible to give. Although Table 1 showed that 7,000 most frequent word families in the BNC/COCA lists would be sufficient to provide 98% coverage for online newspapers and magazines, Figure 1 demonstrated that it was certainly not the case.

Still, if we were to take a broad view to the NOW country sub-corpora and consider Bangladesh as a special case, we could generally conclude that 4,000 and 8,000 most frequent word families in the BNC/COCA lists would reliably provide 95 and 98% coverage of the articles from online newspapers and magazines, which could be a rough answer to research question 1. The findings aligned really well with what Nation found in 2006 with the BNC wordlist. However, it is also worth noting that the 3,000-word level in some cases proved itself to be able to represent 95% coverage for online newspapers and magazines. Most importantly, a considerable proportion of data from the sub-corpora showed that the most frequent 6,000 word families can be a feasible learning goal to rely on. It is obvious that for some countries such as New Zealand, the readers’ vocabulary knowledge only needed to be at the 6,000 level to read online news and magazines for unsupported comprehension.

It is also interesting to compare the results to other researches that also employed the BNC/COCA lists. Specifically, online newspapers and magazines were found to share relatively similar lexical demands with English textbooks for EFL learners (Yang and Coxhead, 2020; Rahmat and Coxhead, 2021) and reading passages in international tests of English proficiency like TOEFL, IELTS, TOIEC, etc. (Kaneko, 2020). This proves that online newspapers and magazines could be a great source for English learners who are preparing for their IELTS or TOEFL tests. However, compared to academic books written in English (Hsu, 2018; Lu and Coxhead, 2020), newspapers were shown to be less lexically demanding, which is normal due to the nature of general and academic English.

Data from Figure 1 as well as Supplementary Appendix, demonstrated a “yes” answer to the second research question. In fact, it was really interesting to see that the most lexically demanding newspapers and magazines came from countries where English was a second or even foreign language like Malaysia, Sri Lanka, Philippines, India, and Bangladesh. On the other hand, in countries where people spoke English as a first language or had native-like English language proficiency such as Canada, Australia, New Zealand, Ireland, United States, United Kingdom, Singapore, and Hong Kong, online newspapers and magazines written in English seemed to be easier to read and understand. Certain explanations could be given to this phenomenon, one of which was the components of the BNC/COCA wordlist. As its name suggested, the corpora that were used to create Nation’s (2017) BNC/COCA frequency lists contained spoken and written texts primarily collected from American and British contexts. As a result, the BNC/COCA lists may have aligned better with the written texts from countries that have been heavily influenced by American and British Englishes. In other words, newspapers and magazines articles that had similar wording patterns to those of American or British written English showed better lexical coverage compared to other countries that had different wording patterns.

The findings would be even more interesting if we were to consider word frequency as an indicator of text difficulty. As Hashimoto (2021) and Stewart et al. (2021) discussed, there was a really strong relationship between word frequency rank and word difficulty. Therefore, it could be said that English newspapers from certain countries may pose greater or lesser challenges to certain English learners. International students and immigrants that have been studying British or American English may find these findings interesting since being able to understand local news could be a great way to establish a sense of connection and belonging to the local communities and networks of a country (Juang et al., 2018).

The study’s results would also of help for English teachers around the world, especially those who are thinking of using English newspapers in their own countries as teaching materials. In fact, articles from online newspapers and magazines could be a great source for English language teaching as they provide up-to-date and interesting information while offering a rich linguistic resources. Using newspapers as reading material could easily trigger learners’ interest and facilitate discussions, especially when they are about hot issues in the country or around the world. Teachers and course book writers would have different criteria when selecting teaching materials. But generally speaking, input resources selected for language learning should be lexically less challenging than what’s in the real world. For example, Tegge (2017) pointed out that songs selected by teachers were 1,000–2,000 word families less demanding than other songs on billboard chart. Collins’s (2017) study also indicated that reading passages in EFL textbooks were significantly easier to read than those appeared in standardized tests of English proficiency. If the lexical demands of the input texts have become the number one concern for lesson and material design, then the most obvious and maybe best practice would be to actively choose articles from English speaking countries including Canada, New Zealand, Australia, and the United Kingdom. Newspapers and magazines from ESL contexts like Hong Kong, Ireland, Jamaica and Tanzania could also be put into consideration when choosing reading materials since their articles showed relatively low lexical demands.

It is also noteworthy that even the lowest figures of lexical demands suggested that a word knowledge at 3,000 level was needed to read online newspaper and magazine articles without having to depend too much on dictionaries. Therefore, it is suggestive that the use of authentic articles from online newspapers and magazines in language courses could only be feasible for upper-intermediate or advanced learners. Language teachers should also make sure that their learners know the 2,000 most frequent word families in the BNC/COCA word list, the knowledge threshold where more than 85% coverage could be guaranteed. This could be done by using vocabulary tests that employed the BNC/COCA lists as the source for test items such as the New Vocabulary Levels Test (McLean and Kramer, 2015) the Listening Vocabulary Levels Test (McLean et al., 2015; Ha, 2021a) and Webb et al.’s (2017) Updated Vocabulary Levels Test. The vocabulary knowledge at 2,000 level generally ensures that learners could at least work with the material, of course with the support from teachers and/or more capable peers.

English teachers of advanced classes may use up-to-date newspaper articles as in-class reading activities where learners together read an interesting article and then discuss it. Language instructors can also assign learners to pick articles of their interest that reflect current situations around the world to read extensively, and then discuss what they have read with their peers when they come back to the class. Such practices might be especially suitable for English teachers of immigrants and refugees who would be in dire need of both the language and the updated information of the countries where they were currently based. However, it might be somewhat unrealistic to expect most immigrants and refugees to have knowledge of the most frequent 2,000 word families in the BNC/COCA word list.

## Limitations

The present study bears certain limitations that need to be addressed. As the study employed lexical profiler program accompanied by designed wordlists as the primary research methodology, it was unavoidably affected by the limitations of such approach (Nation and Webb, 2011).

The first limitation was the inability of lexical profiler programs such as RANGE to identify homographs [e.g., *proceeds* (meaning *continues*) and *proceeds* (meaning *profits*)]. Such a constraint may also have affected the classification of proper nouns since certain proper nouns bear the same spelling as other words (*Gates, Walkers, Bush*, etc.), leading to the difficult situation where manually adding these words to the list of proper nouns would cause severe conflicts in processing, and leaving them alone would result in these words being ranked as high-frequency words.

Second, lexical profiler programs such as RANGE could not count multiword items as single items. This was, in my opinion, one of the most serious flaws which most lexical profiling research that utilized the same research methodology have been suffering. As the classification of RANGE and other programs like AntWordProfiler was guided by word lists that contained primarily single-item words, they would read phrasal verbs and idioms such as *out of the blue, out of the box, sleep on it, come across, sit up…* separately and rank the components words of these phrases according to their designed frequency levels. Although most the component words of phrasal verbs and idioms are high-frequency verbs, understanding every single item in such phrases could not guarantee the comprehension of the phrase as a whole (Cornell, 1985; Gardner and Davies, 2007; Garnier and Schmitt, 2015).

The third point that deserves attention concerned how transparent and hyphenated compounds were treated. The present study adopted two assumptions that have been widely applied in vocabulary profiling research (Dang and Webb, 2014; Nurmukhamedov, 2017; Tegge, 2017; Yang and Coxhead, 2020; Nurmukhamedov and Sharakhimov, 2021; Rahmat and Coxhead, 2021) that transparent and hyphenated compounds could be easily understood by knowing the meaning of their component words, which could be problematic to certain extents. For example, whether or not transparent compounds such as *aftershock*, *afterglow* or *absentminded* could be understood by the sheer knowledge of *after, shock, glow*, and *mind* was actually a myth. Similarly, assuming a learner could understand *sale-driven* based on his or her knowledge of *driven* in the sentence: “*The car is driven by Jack.”* could be also be a questionable practice.

Besides the limitations concerning research methodology, there was another area that the present study could not address. Although the manuscript showed strong variations in the lexical demands between different countries, it only provided statistical arguments and did not address the issue from a socio-linguistic perspective. Therefore, future studies are encouraged to explore the link between cultural and social-economic factors of countries and their publications’ lexical profile.

## Conclusion

The present study offers insights into the vocabulary load of the most popular sources of written English that people read every day. Its findings indicate that knowledge of the most frequent 3,000–4,000 word families plus proper nouns, marginal words, transparent compounds and acronyms could provide 95% coverage of the articles in online newspapers and magazines, which might be a degree of coverage for adequate comprehension and incidental vocabulary learning.

The study confirms Nation’s (2006) findings but emphasizes that coverage of newspaper and magazine articles varies greatly between countries, and that articles from English speaking countries are less lexically demanding than those in ESL and EFL contexts. The results also suggest that web-based newspapers and magazines could be good resources for language teaching and learning. However, it is advised that teachers and learners need to be very selective when choosing what to read as articles from certain countries were shown to be relatively more difficult to understand than others.

## Data Availability Statement

The data analyzed in this study is subject to the following licenses/restrictions: the corpora that support the findings of this study are available from Mark Davies. Restrictions apply to the availability of these corpora, which were used under academic license for this study. Data are available from https://www.english-corpora.org/ with the permission of Mark Davies. Requests to access these datasets should be directed to mark.davies@corpusdata.org.

## Author Contributions

The author confirms sole responsibility for study conception and design, data collection, analysis and interpretation of results, and manuscript preparation.

## Conflict of Interest

The author declares that the research was conducted in the absence of any commercial or financial relationships that could be construed as a potential conflict of interest.

## Publisher’s Note

All claims expressed in this article are solely those of the authors and do not necessarily represent those of their affiliated organizations, or those of the publisher, the editors and the reviewers. Any product that may be evaluated in this article, or claim that may be made by its manufacturer, is not guaranteed or endorsed by the publisher.

## Footnotes

1
https://www.english-corpora.org/

## Abbreviations

BNC: British National Corpus
COCA: Corpus of Contemporary American English
ESL: English as a second language
EFL: English as a foreign language
MW: marginal words
NOW: News on the Web
PN: proper nouns
TC: transparent compounds
US: United States
UK: United Kingdom.
