# Supplementary material for: Lexical Profile of Newspapers Revisited: A Corpus-Based Analysis
Source: Front Psychol. 2022 Feb 24;13:800983. doi: 10.3389/fpsyg.2022.800983 (PMC8907927; doi:10.3389/fpsyg.2022.800983)
Supplement: Supplementary file 1 [file Table_1.docx]

Appendix A

**Supplementary Table 1. Cumulative coverage including proper nouns, marginal words, transparent compounds and acronyms for each NOW sub-corpus.**

| US | | | | | | | | | | | | |
| --- | --- | --- | --- | --- | --- | --- | --- | --- | --- | --- | --- | --- |
| Word list | 2010 | 2011 | 2012 | 2013 | 2014 | 2015 | 2016 | 2017 | 2018 | 2019 | 2020 | 2021 |
| 1,000 | 79.13 | 79.06 | 78.94 | 78.68 | 78.62 | 78.90 | 79.25 | 79.25 | 78.95 | 78.16 | 77.54 | 77.48 |
| 2,000 | 88.60 | 88.59 | 88.51 | 88.33 | 88.29 | 88.47 | 88.90 | 88.95 | 88.74 | 88.31 | 87.92 | 87.67 |
| 3,000 | 94.30 | 94.35 | 94.37 | 94.32 | 94.35 | 94.39 | 94.45 | 94.44 | 94.38 | 94.08 | 94.15 | 94.02 |
| 4,000 | **96.11** | **96.13** | **96.15** | **96.12** | **96.15** | **96.17** | **96.18** | **96.19** | **96.15** | **95.91** | **95.95** | **95.82** |
| 5,000 | 97.10 | 97.12 | 97.13 | 97.12 | 97.15 | 97.16 | 97.16 | 97.17 | 97.15 | 96.93 | 96.94 | 96.86 |
| 6,000 | 97.77 | 97.79 | 97.79 | 97.79 | 97.82 | 97.83 | 97.89 | **97.90** | 97.85 | 97.63 | 97.64 | 97.62 |
| 7,000 | **98.23** | **98.25** | **98.26** | **98.26** | **98.29** | **98.29** | **98.33** | **98.34** | **98.29** | **98.09** | **98.07** | **98.06** |
| 8,000 | 98.64 | 98.65 | 98.66 | 98.65 | 98.67 | 98.68 | 98.73 | 98.75 | 98.69 | 98.54 | 98.48 | 98.46 |
| 9,000 | 98.88 | 98.90 | 98.91 | 98.90 | 98.92 | 98.93 | 98.97 | 98.97 | 98.93 | 98.79 | 98.71 | 98.71 |
| 10,000 – 25,000 | 99.76 | 99.76 | 99.77 | 99.77 | 99.77 | 99.75 | 99.73 | 99.72 | 99.70 | 99.65 | 99.67 | 99.66 |
| Not in the lists | 100 | 100 | 100 | 100 | 100 | 100 | 100 | 100 | 100 | 100 | 100 | 100 |
| UK | | | | | | | | | | | | |
| Word list | 2010 | 2011 | 2012 | 2013 | 2014 | 2015 | 2016 | 2017 | 2018 | 2019 | 2020 | 2021 |
| 1,000 | 79.95 | 79.76 | 79.85 | 79.73 | 79.65 | 79.70 | 79.98 | 79.97 | 79.68 | 79.51 | 78.88 | 77.87 |
| 2,000 | 89.22 | 89.09 | 89.16 | 89.16 | 89.03 | 89.05 | 89.49 | 89.50 | 89.40 | 89.31 | 89.03 | 88.40 |
| 3,000 | 94.52 | 94.46 | 94.49 | 94.56 | 94.50 | 94.49 | 94.81 | 94.84 | 94.86 | 94.83 | 94.85 | 94.71 |
| 4,000 | **96.25** | **96.20** | **96.23** | **96.28** | **96.22** | **96.22** | **96.44** | **96.46** | **96.48** | **96.48** | **96.52** | **96.39** |
| 5,000 | 97.20 | 97.18 | 97.19 | 97.24 | 97.21 | 97.21 | 97.37 | 97.39 | 97.42 | 97.42 | 97.42 | 97.36 |
| 6,000 | 97.91 | 97.87 | 97.87 | 97.90 | 97.87 | 97.88 | **98.04** | **98.08** | **98.07** | **98.06** | **98.04** | **98.02** |
| 7,000 | **98.36** | **98.34** | **98.34** | **98.36** | **98.33** | **98.34** | 98.46 | 98.47 | 98.48 | 98.47 | 98.41 | 98.42 |
| 8,000 | 98.74 | 98.73 | 98.73 | 98.74 | 98.72 | 98.72 | 98.82 | 98.83 | 98.83 | 98.83 | 98.75 | 98.76 |
| 9,000 | 98.99 | 98.98 | 98.99 | 99.00 | 98.99 | 98.98 | 99.06 | 99.06 | 99.06 | 99.06 | 98.97 | 98.98 |
| 10,000 – 25,000 | 99.80 | 99.80 | 99.80 | 99.80 | 99.79 | 99.78 | 99.77 | 99.76 | 99.76 | 99.76 | 99.76 | 99.74 |
| Not in the lists | 100 | 100 | 100 | 100 | 100 | 100 | 100 | 100 | 100 | 100 | 100 | 100 |
| Tanzania | | | | | | | | | | | | |
| Word list | 2010 | 2011 | 2012 | 2013 | 2014 | 2015 | 2016 | 2017 | 2018 | 2019 | 2020 | 2021 |
| 1,000 | 74.16 | 74.99 | 74.54 | 73.92 | 73.47 | 73.97 | 74.10 | 70.97 | 74.84 | 75.57 | 74.08 | 75.03 |
| 2,000 | 86.39 | 87.09 | 86.87 | 86.45 | 86.27 | 86.43 | 86.53 | 84.94 | 87.17 | 87.37 | 86.41 | 86.98 |
| 3,000 | **95.14** | 94.88 | **94.94** | **94.99** | **94.99** | 94.87 | 94.73 | 93.89 | **95.10** | **95.01** | 94.71 | 94.86 |
| 4,000 | 96.59 | **96.62** | 96.70 | 96.76 | 96.66 | **96.61** | **96.49** | **96.03** | 96.76 | 96.68 | **96.50** | **96.55** |
| 5,000 | 97.33 | 97.57 | 97.56 | 97.63 | 97.51 | 97.46 | 97.31 | 97.03 | 97.58 | 97.55 | 97.42 | 97.48 |
| 6,000 | 97.88 | **98.15** | **98.10** | **98.19** | **98.05** | **97.97** | 97.83 | 97.66 | **98.12** | **98.11** | **98.01** | **98.08** |
| 7,000 | **98.39** | 98.51 | 98.48 | 98.58 | 98.47 | 98.50 | **98.38** | **97.97** | 98.45 | 98.46 | 98.37 | 98.44 |
| 8,000 | 98.72 | 98.83 | 98.77 | 98.86 | 98.77 | 98.79 | 98.69 | 98.36 | 98.77 | 98.79 | 98.67 | 98.73 |
| 9,000 | 98.85 | 98.99 | 98.94 | 99.02 | 98.92 | 98.95 | 98.91 | 98.53 | 98.97 | 98.98 | 98.86 | 98.91 |
| 10,000 – 25,000 | 99.56 | 99.49 | 99.47 | 99.50 | 99.48 | 99.48 | 99.52 | 99.42 | 99.53 | 99.52 | 99.55 | 99.51 |
| Not in the lists | 100 | 100 | 100 | 100 | 100 | 100 | 100 | 100 | 100 | 100 | 100 | 100 |
| Sri Lanka | | | | | | | | | | | | |
| Word list | 2010 | 2011 | 2012 | 2013 | 2014 | 2015 | 2016 | 2017 | 2018 | 2019 | 2020 | 2021 |
| 1,000 | 76.51 | 76.09 | 77.40 | 75.64 | 76.00 | 75.47 | 75.58 | 75.00 | 74.44 | 74.22 | 73.86 | 74.00 |
| 2,000 | 87.43 | 86.69 | 87.88 | 86.82 | 87.01 | 87.06 | 87.02 | 86.68 | 86.43 | 86.35 | 86.07 | 85.83 |
| 3,000 | 94.02 | 93.39 | 94.28 | 94.08 | 94.27 | 94.55 | 94.56 | 94.38 | 94.18 | 94.26 | 94.26 | 93.94 |
| 4,000 | **95.84** | **95.11** | **96.00** | **95.88** | **95.97** | **96.22** | **96.25** | **96.11** | **95.92** | **96.01** | **96.04** | **95.77** |
| 5,000 | 96.94 | 96.20 | 97.12 | 97.11 | 97.07 | 97.26 | 97.31 | 97.17 | 96.99 | 97.11 | 97.08 | 96.96 |
| 6,000 | 97.64 | 96.86 | 97.76 | 97.71 | 97.70 | 97.85 | 97.93 | 97.84 | 97.67 | 97.78 | 97.74 | 97.69 |
| 7,000 | **98.09** | 97.29 | **98.17** | **98.12** | **98.11** | **98.25** | **98.34** | **98.26** | **98.09** | **98.21** | **98.15** | **98.12** |
| 8,000 | 98.48 | **98.54** | 98.53 | 98.51 | 98.48 | 98.60 | 98.66 | 98.62 | 98.49 | 98.60 | 98.55 | 98.50 |
| 9,000 | 98.71 | 98.78 | 98.75 | 98.73 | 98.71 | 98.81 | 98.89 | 98.86 | 98.76 | 98.87 | 98.82 | 98.78 |
| 10,000 – 25,000 | 99.44 | 99.48 | 99.46 | 99.44 | 99.44 | 99.48 | 99.55 | 99.55 | 99.55 | 99.57 | 99.60 | 99.58 |
| Not in the lists | 100 | 100 | 100 | 100 | 100 | 100 | 100 | 100 | 100 | 100 | 100 | 100 |
| South Africa | | | | | | | | | | | | |
| Word list | 2010 | 2011 | 2012 | 2013 | 2014 | 2015 | 2016 | 2017 | 2018 | 2019 | 2020 | 2021 |
| 1,000 | 78.88 | 78.37 | 77.59 | 77.70 | 78.42 | 78.05 | 78.07 | 77.80 | 77.43 | 77.07 | 76.24 | 75.82 |
| 2,000 | 88.71 | 88.47 | 88.16 | 88.26 | 88.59 | 88.44 | 88.41 | 88.24 | 88.04 | 87.89 | 87.37 | 87.07 |
| 3,000 | 94.60 | 94.40 | 94.34 | 94.39 | 94.58 | 94.51 | 94.64 | 94.64 | 94.60 | 94.62 | 94.43 | 94.20 |
| 4,000 | **96.33** | **96.18** | **96.29** | **96.33** | **96.33** | **96.36** | **96.36** | **96.36** | **96.34** | **96.37** | **96.22** | **96.02** |
| 5,000 | 97.21 | 97.12 | 97.23 | 97.26 | 97.21 | 97.29 | 97.26 | 97.26 | 97.24 | 97.27 | 97.13 | 97.02 |
| 6,000 | 97.82 | 97.78 | 97.86 | 97.89 | 97.82 | 97.89 | 97.86 | 97.86 | 97.86 | 97.89 | 97.77 | 97.72 |
| 7,000 | **98.17** | **98.16** | **98.25** | **98.27** | **98.24** | **98.30** | **98.27** | **98.29** | **98.28** | **98.29** | **98.22** | **98.16** |
| 8,000 | 98.55 | 98.55 | 98.63 | 98.65 | 98.63 | 98.67 | 98.65 | 98.65 | 98.63 | 98.63 | 98.57 | 98.52 |
| 9,000 | 98.78 | 98.78 | 98.91 | 98.92 | 98.85 | 98.93 | 98.89 | 98.89 | 98.86 | 98.86 | 98.79 | 98.75 |
| 10,000 – 25,000 | 99.51 | 99.54 | 99.57 | 99.57 | 99.55 | 99.59 | 99.55 | 99.53 | 99.53 | 99.53 | 99.58 | 99.54 |
| Not in the lists | 100 | 100 | 100 | 100 | 100 | 100 | 100 | 100 | 100 | 100 | 100 | 100 |
| Singapore | | | | | | | | | | | | |
| Word list | 2010 | 2011 | 2012 | 2013 | 2014 | 2015 | 2016 | 2017 | 2018 | 2019 | 2020 | 2021 |
| 1,000 | 78.86 | 76.72 | 75.58 | 75.96 | 76.16 | 75.87 | 76.48 | 76.31 | 76.36 | 76.32 | 76.05 | 75.77 |
| 2,000 | 88.49 | 87.54 | 86.77 | 86.98 | 87.17 | 87.24 | 87.48 | 87.34 | 87.42 | 87.37 | 87.05 | 86.81 |
| 3,000 | 94.22 | 94.02 | 93.85 | 93.81 | 94.17 | 94.32 | 94.33 | 94.26 | 94.40 | 94.40 | 94.27 | 94.03 |
| 4,000 | **95.89** | **95.84** | **95.77** | **95.84** | **96.11** | **96.24** | **96.23** | **96.13** | **96.18** | **96.18** | **96.01** | **95.83** |
| 5,000 | 96.95 | 96.85 | 96.83 | 96.86 | 97.03 | 97.15 | 97.15 | 97.06 | 97.12 | 97.12 | 96.96 | 96.87 |
| 6,000 | 97.68 | 97.52 | 97.53 | 97.58 | 97.77 | 97.84 | 97.88 | 97.83 | 97.82 | 97.82 | 97.67 | 97.60 |
| 7,000 | **98.09** | **97.91** | **97.94** | **97.99** | **98.16** | **98.22** | **98.26** | **98.23** | **98.22** | **98.23** | **98.07** | **98.02** |
| 8,000 | 98.45 | 98.32 | 98.39 | 98.42 | 98.58 | 98.63 | 98.67 | 98.65 | 98.63 | 98.62 | 98.46 | 98.42 |
| 9,000 | 98.71 | 98.53 | 98.62 | 98.66 | 98.81 | 98.84 | 98.87 | 98.87 | 98.85 | 98.84 | 98.68 | 98.65 |
| 10,000 – 25,000 | 99.61 | 99.51 | 99.54 | 99.58 | 99.60 | 99.58 | 99.59 | 99.59 | 99.58 | 99.59 | 99.60 | 99.57 |
| Not in the lists | 100 | 100 | 100 | 100 | 100 | 100 | 100 | 100 | 100 | 100 | 100 | 100 |
| Philippines | | | | | | | | | | | | |
| Word list | 2010 | 2011 | 2012 | 2013 | 2014 | 2015 | 2016 | 2017 | 2018 | 2019 | 2020 | 2021 |
| 1,000 | 77.55 | 78.12 | 77.29 | 76.66 | 76.05 | 76.30 | 76.12 | 76.32 | 76.89 | 77.34 | 77.09 | 75.58 |
| 2,000 | 87.16 | 87.75 | 87.35 | 87.03 | 86.76 | 86.91 | 86.84 | 86.74 | 87.23 | 87.55 | 87.47 | 86.41 |
| 3,000 | 93.13 | 93.64 | 93.71 | 93.68 | 93.65 | 93.74 | 93.78 | 93.59 | 93.76 | 93.98 | 93.96 | 93.53 |
| 4,000 | **95.18** | **95.50** | **95.58** | **95.56** | **95.55** | **95.62** | **95.65** | **95.49** | **95.59** | **95.76** | **95.70** | **95.35** |
| 5,000 | 96.31 | 96.56 | 96.65 | 96.67 | 96.68 | 96.74 | 96.76 | 96.60 | 96.69 | 96.82 | 96.74 | 96.53 |
| 6,000 | 97.03 | 97.29 | 97.37 | 97.37 | 97.37 | 97.44 | 97.44 | 97.33 | 97.41 | 97.51 | 97.39 | 97.26 |
| 7,000 | 97.49 | 97.77 | 97.81 | 97.81 | 97.79 | 97.86 | 97.88 | 97.80 | 97.86 | **97.94** | 97.80 | 97.69 |
| 8,000 | **97.96** | **98.18** | **98.22** | **98.22** | **98.21** | **98.28** | **98.28** | **98.26** | **98.27** | 98.35 | **98.17** | **98.12** |
| 9,000 | 98.22 | 98.45 | 98.49 | 98.50 | 98.51 | 98.56 | 98.57 | 98.56 | 98.54 | 98.61 | 98.49 | 98.44 |
| 10,000 – 25,000 | 99.23 | 99.40 | 99.39 | 99.36 | 99.33 | 99.37 | 99.36 | 99.34 | 99.37 | 99.40 | 99.42 | 99.36 |
| Not in the lists | 100 | 100 | 100 | 100 | 100 | 100 | 100 | 100 | 100 | 100 | 100 | 100 |
| Pakistan | | | | | | | | | | | | |
| Word list | 2010 | 2011 | 2012 | 2013 | 2014 | 2015 | 2016 | 2017 | 2018 | 2019 | 2020 | 2021 |
| 1,000 | 77.60 | 77.92 | 77.34 | 76.85 | 76.35 | 76.10 | 75.20 | 76.32 | 75.44 | 75.05 | 75.14 | 74.05 |
| 2,000 | 87.87 | 88.07 | 87.77 | 87.53 | 87.12 | 87.00 | 86.60 | 86.74 | 86.75 | 86.58 | 86.46 | 85.86 |
| 3,000 | 94.25 | 94.30 | 94.33 | 94.30 | 94.19 | 94.22 | 94.31 | 93.59 | 94.27 | 94.30 | 94.04 | 93.88 |
| 4,000 | **95.97** | **95.96** | **96.02** | **96.01** | **95.95** | **95.96** | **96.06** | **95.49** | **96.01** | **96.02** | **95.77** | **95.66** |
| 5,000 | 96.95 | 96.95 | 97.02 | 97.00 | 96.94 | 96.97 | 97.06 | 96.60 | 97.00 | 97.01 | 96.83 | 96.77 |
| 6,000 | 97.58 | 97.57 | 97.65 | 97.62 | 97.58 | 97.61 | 97.71 | 97.33 | 97.63 | 97.66 | 97.47 | 97.47 |
| 7,000 | **98.04** | **98.02** | **98.08** | **98.06** | **98.03** | **98.05** | **98.12** | **97.80** | **98.06** | **98.07** | **97.88** | **97.90** |
| 8,000 | 98.41 | 98.40 | 98.45 | 98.43 | 98.42 | 98.42 | 98.49 | 98.26 | 98.41 | 98.43 | 98.24 | 98.27 |
| 9,000 | 98.63 | 98.63 | 98.67 | 98.65 | 98.66 | 98.65 | 98.73 | 98.56 | 98.64 | 98.67 | 98.50 | 98.53 |
| 10,000 – 25,000 | 99.49 | 99.48 | 99.49 | 99.48 | 99.48 | 99.48 | 99.51 | 99.34 | 99.45 | 99.48 | 99.47 | 99.50 |
| Not in the lists | 100 | 100 | 100 | 100 | 100 | 100 | 100 | 100 | 100 | 100 | 100 | 100 |
| Nigeria | | | | | | | | | | | | |
| Word list | 2010 | 2011 | 2012 | 2013 | 2014 | 2015 | 2016 | 2017 | 2018 | 2019 | 2020 | 2021 |
| 1,000 | 78.78 | 77.76 | 77.51 | 78.06 | 77.45 | 76.77 | 77.28 | 76.64 | 75.98 | 75.96 | 75.87 | 75.18 |
| 2,000 | 88.30 | 87.66 | 87.58 | 87.87 | 87.45 | 87.09 | 87.49 | 87.18 | 86.82 | 86.75 | 86.54 | 85.93 |
| 3,000 | 94.44 | 94.29 | 94.30 | 94.29 | 94.19 | 94.02 | 94.23 | 94.28 | 94.25 | 94.16 | 93.95 | 93.52 |
| 4,000 | **96.21** | **96.09** | **96.07** | **96.05** | **95.99** | **95.88** | **96.05** | **96.07** | **96.09** | **96.05** | **95.79** | **95.44** |
| 5,000 | 97.19 | 97.09 | 97.06 | 97.03 | 96.98 | 96.93 | 97.09 | 97.09 | 97.08 | 97.05 | 96.82 | 96.58 |
| 6,000 | 97.79 | 97.70 | 97.69 | 97.67 | 97.65 | 97.61 | 97.75 | 97.76 | 97.74 | 97.69 | 97.50 | 97.34 |
| 7,000 | **98.22** | **98.14** | **98.13** | **98.11** | **98.10** | **98.06** | **98.16** | **98.17** | **98.14** | **98.11** | **97.96** | **97.95** |
| 8,000 | 98.56 | 98.46 | 98.47 | 98.46 | 98.44 | 98.40 | 98.50 | 98.49 | 98.50 | 98.44 | 98.30 | 98.33 |
| 9,000 | 98.78 | 98.72 | 98.75 | 98.73 | 98.72 | 98.68 | 98.74 | 98.73 | 98.74 | 98.69 | 98.57 | 98.59 |
| 10,000 – 25,000 | 99.53 | 99.48 | 99.49 | 99.50 | 99.49 | 99.49 | 99.47 | 99.46 | 99.44 | 99.44 | 99.46 | 99.43 |
| Not in the lists | 100 | 100 | 100 | 100 | 100 | 100 | 100 | 100 | 100 | 100 | 100 | 100 |
| New Zealand | | | | | | | | | | | | |
| Word list | 2010 | 2011 | 2012 | 2013 | 2014 | 2015 | 2016 | 2017 | 2018 | 2019 | 2020 | 2021 |
| 1,000 | 79.65 | 80.24 | 80.63 | 80.39 | 80.17 | 80.20 | 79.94 | 80.15 | 79.80 | 79.42 | 78.84 | 78.14 |
| 2,000 | 89.32 | 89.67 | 89.94 | 89.85 | 89.88 | 89.93 | 89.68 | 89.79 | 89.55 | 89.33 | 88.97 | 88.53 |
| 3,000 | 94.70 | 94.75 | 94.88 | **94.92** | **95.01** | **95.04** | **94.97** | **95.05** | **95.02** | **94.97** | **95.17** | **94.92** |
| 4,000 | **96.39** | **96.39** | **96.50** | 96.53 | 96.60 | 96.60 | 96.54 | 96.58 | 96.58 | 96.55 | 96.69 | 96.51 |
| 5,000 | 97.36 | 97.37 | 97.44 | 97.46 | 97.54 | 97.53 | 97.48 | 97.52 | 97.52 | 97.48 | 97.57 | 97.48 |
| 6,000 | **98.01** | **98.05** | **98.08** | **98.09** | **98.13** | **98.13** | **98.11** | **98.16** | **98.15** | **98.13** | **98.18** | **98.13** |
| 7,000 | 98.40 | 98.46 | 98.49 | 98.47 | 98.50 | 98.49 | 98.49 | 98.51 | 98.52 | 98.50 | 98.51 | 98.48 |
| 8,000 | 98.71 | 98.76 | 98.79 | 98.78 | 98.79 | 98.80 | 98.80 | 98.82 | 98.83 | 98.81 | 98.82 | 98.78 |
| 9,000 | 98.97 | 99.02 | 99.05 | 99.05 | 99.05 | 99.06 | 99.07 | 99.08 | 99.08 | 99.06 | 99.06 | 99.03 |
| 10,000 – 25,000 | 99.74 | 99.75 | 99.77 | 99.76 | 99.74 | 99.73 | 99.75 | 99.76 | 99.75 | 99.75 | 99.75 | 99.72 |
| Not in the lists | 100 | 100 | 100 | 100 | 100 | 100 | 100 | 100 | 100 | 100 | 100 | 100 |
| Malaysia | | | | | | | | | | | | |
| Word list | 2010 | 2011 | 2012 | 2013 | 2014 | 2015 | 2016 | 2017 | 2018 | 2019 | 2020 | 2021 |
| 1,000 | 80.11 | 78.72 | 77.32 | 77.13 | 77.56 | 77.30 | 76.90 | 74.69 | 76.20 | 77.12 | 77.85 | 75.85 |
| 2,000 | 89.48 | 88.84 | 88.14 | 87.99 | 87.99 | 87.83 | 87.82 | 87.04 | 87.44 | 87.82 | 88.22 | 86.93 |
| 3,000 | 94.02 | 94.21 | 94.38 | 94.40 | 94.35 | 94.36 | 94.57 | 94.43 | 94.49 | 94.45 | 94.59 | 94.20 |
| 4,000 | **95.54** | **95.88** | **95.98** | **96.01** | **96.00** | **95.96** | **96.26** | **96.03** | **96.19** | **96.14** | **96.20** | **95.90** |
| 5,000 | 96.43 | 96.83 | 96.94 | 97.00 | 97.00 | 96.95 | 97.18 | 96.82 | 97.09 | 97.06 | 97.09 | 96.98 |
| 6,000 | 97.08 | 97.48 | 97.55 | 97.62 | 97.63 | 97.57 | 97.78 | 97.38 | 97.71 | 97.69 | 97.71 | 97.65 |
| 7,000 | 97.78 | 97.89 | **97.98** | **98.02** | **98.06** | **98.03** | **98.15** | 97.69 | **98.07** | **98.08** | **98.06** | **98.00** |
| 8,000 | **98.18** | **98.26** | 98.36 | 98.38 | 98.40 | 98.37 | 98.50 | **98.25** | 98.43 | 98.42 | 98.39 | 98.35 |
| 9,000 | 98.42 | 98.55 | 98.63 | 98.63 | 98.65 | 98.62 | 98.70 | 98.44 | 98.63 | 98.62 | 98.58 | 98.55 |
| 10,000 – 25,000 | 99.45 | 99.51 | 99.46 | 99.44 | 99.49 | 99.47 | 99.44 | 99.23 | 99.37 | 99.39 | 99.43 | 99.39 |
| Not in the lists | 100 | 100 | 100 | 100 | 100 | 100 | 100 | 100 | 100 | 100 | 100 | 100 |
| Kenya | | | | | | | | | | | | |
| Word list | 2010 | 2011 | 2012 | 2013 | 2014 | 2015 | 2016 | 2017 | 2018 | 2019 | 2020 | 2021 |
| 1,000 | 74.77 | 78.37 | 76.09 | 76.33 | 76.06 | 76.67 | 75.73 | 76.72 | 76.97 | 77.23 | 76.73 | 75.90 |
| 2,000 | 86.17 | 88.30 | 87.44 | 87.46 | 87.25 | 87.52 | 87.66 | 87.55 | 87.60 | 87.83 | 87.46 | 86.81 |
| 3,000 | 94.11 | 94.58 | 94.44 | 94.39 | 94.27 | 94.38 | 94.71 | 94.29 | 94.25 | 94.37 | 94.28 | 93.95 |
| 4,000 | **96.03** | **96.15** | **96.15** | **96.12** | **95.99** | **96.08** | **96.48** | **96.07** | **96.03** | **96.14** | **96.03** | **95.73** |
| 5,000 | 97.23 | 97.07 | 97.11 | 97.09 | 96.93 | 97.03 | 97.27 | 97.06 | 97.05 | 97.07 | 96.97 | 96.76 |
| 6,000 | 97.79 | 97.69 | 97.76 | 97.75 | 97.62 | 97.72 | 97.81 | 97.69 | 97.69 | 97.70 | 97.62 | 97.49 |
| 7,000 | **98.17** | **98.12** | **98.17** | **98.16** | **98.02** | **98.13** | **98.13** | **98.10** | **98.12** | **98.12** | **98.06** | **98.15** |
| 8,000 | 98.54 | 98.53 | 98.55 | 98.56 | 98.55 | 98.55 | 98.63 | 98.58 | 98.52 | 98.51 | 98.45 | 98.56 |
| 9,000 | 98.80 | 98.75 | 98.82 | 98.81 | 98.82 | 98.83 | 98.85 | 98.84 | 98.80 | 98.77 | 98.72 | 98.80 |
| 10,000 – 25,000 | 99.58 | 99.45 | 99.53 | 99.54 | 99.54 | 99.53 | 99.44 | 99.48 | 99.44 | 99.44 | 99.51 | 99.49 |
| Not in the lists | 100 | 100 | 100 | 100 | 100 | 100 | 100 | 100 | 100 | 100 | 100 | 100 |
| Jamaica | | | | | | | | | | | | |
| Word list | 2010 | 2011 | 2012 | 2013 | 2014 | 2015 | 2016 | 2017 | 2018 | 2019 | 2020 | 2021 |
| 1,000 | 78.55 | 78.51 | 78.63 | 77.52 | 77.19 | 77.28 | 78.57 | 79.23 | 77.68 | 76.21 | 75.94 | 77.44 |
| 2,000 | 88.83 | 88.70 | 88.78 | 88.46 | 88.30 | 88.41 | 89.48 | 89.36 | 88.49 | 87.75 | 87.74 | 89.05 |
| 3,000 | 94.64 | 94.76 | 94.75 | **94.94** | **95.00** | **95.16** | **95.40** | **95.10** | **94.90** | **95.20** | **95.31** | **95.34** |
| 4,000 | **96.29** | **96.44** | **96.42** | 96.55 | 96.55 | 96.71 | 96.80 | 96.66 | **96.62** | 96.86 | 96.87 | 96.63 |
| 5,000 | 97.24 | 97.39 | 97.34 | 97.43 | 97.41 | 97.56 | 97.60 | 97.50 | 97.49 | 97.69 | 97.66 | 97.49 |
| 6,000 | 97.88 | **98.00** | **97.96** | **98.04** | **98.03** | **98.16** | **98.12** | **98.10** | **98.12** | **98.28** | **98.17** | **98.04** |
| 7,000 | **98.43** | 98.49 | 98.47 | 98.55 | 98.56 | 98.64 | 98.60 | 98.55 | 98.49 | 98.58 | 98.48 | 98.45 |
| 8,000 | 98.77 | 98.82 | 98.81 | 98.84 | 98.85 | 98.92 | 98.85 | 98.83 | 98.79 | 98.85 | 98.74 | 98.68 |
| 9,000 | 99.06 | 99.08 | 99.09 | 99.14 | 99.15 | 99.20 | 99.20 | 99.12 | 99.06 | 99.09 | 98.98 | 98.99 |
| 10,000 – 25,000 | 99.80 | 99.81 | 99.82 | 99.83 | 99.81 | 99.82 | 99.81 | 99.78 | 99.69 | 99.71 | 99.71 | 99.72 |
| Not in the lists | 100 | 100 | 100 | 100 | 100 | 100 | 100 | 100 | 100 | 100 | 100 | 100 |
| Ireland | | | | | | | | | | | | |
| Word list | 2010 | 2011 | 2012 | 2013 | 2014 | 2015 | 2016 | 2017 | 2018 | 2019 | 2020 | 2021 |
| 1,000 | 80.85 | 80.78 | 80.92 | 81.15 | 80.99 | 80.79 | 80.94 | 80.69 | 79.90 | 79.74 | 79.35 | 79.00 |
| 2,000 | 89.82 | 89.63 | 89.79 | 89.95 | 89.95 | 89.83 | 89.92 | 89.89 | 89.37 | 89.37 | 89.19 | 88.97 |
| 3,000 | 94.82 | 94.72 | 94.77 | 94.81 | 94.89 | **94.91** | **95.08** | **95.15** | **95.01** | **94.98** | **94.90** | 94.77 |
| 4,000 | **96.41** | **96.26** | **96.32** | **96.35** | **96.45** | 96.45 | 96.50 | 96.56 | 96.48 | 96.49 | **96.46** | **96.38** |
| 5,000 | 97.28 | 97.20 | 97.21 | 97.20 | 97.30 | 97.33 | 97.34 | 97.38 | 97.36 | 97.37 | 97.32 | 97.30 |
| 6,000 | 97.86 | 97.80 | 97.77 | 97.77 | 97.86 | **97.91** | **97.91** | **97.95** | **97.93** | **97.95** | **97.91** | **97.93** |
| 7,000 | **98.48** | **98.44** | **98.40** | **98.41** | **98.46** | 98.50 | 98.42 | 98.44 | 98.45 | 98.44 | 98.40 | 98.43 |
| 8,000 | 98.80 | 98.75 | 98.72 | 98.72 | 98.77 | 98.81 | 98.76 | 98.75 | 98.75 | 98.76 | 98.73 | 98.75 |
| 9,000 | 99.03 | 99.03 | 98.99 | 98.98 | 99.01 | 99.05 | 99.07 | 99.07 | 99.03 | 99.03 | 98.97 | 99.00 |
| 10,000 – 25,000 | 99.79 | 99.78 | 99.78 | 99.77 | 99.77 | 99.78 | 99.76 | 99.74 | 99.69 | 99.70 | 99.70 | 99.69 |
| Not in the lists | 100 | 100 | 100 | 100 | 100 | 100 | 100 | 100 | 100 | 100 | 100 | 100 |
| India | | | | | | | | | | | | |
| Word list | 2010 | 2011 | 2012 | 2013 | 2014 | 2015 | 2016 | 2017 | 2018 | 2019 | 2020 | 2021 |
| 1,000 | 77.36 | 77.68 | 77.59 | 76.88 | 77.14 | 76.75 | 76.31 | 76.10 | 75.56 | 75.77 | 75.67 | 74.97 |
| 2,000 | 87.56 | 87.81 | 87.77 | 87.33 | 87.47 | 87.20 | 86.95 | 86.83 | 86.57 | 86.69 | 86.53 | 86.03 |
| 3,000 | 93.82 | 93.91 | 93.94 | 93.84 | 93.75 | 93.63 | 93.57 | 93.58 | 93.45 | 93.72 | 93.67 | 93.31 |
| 4,000 | **95.65** | **95.70** | **95.74** | **95.70** | **95.56** | **95.47** | **95.44** | **95.44** | **95.33** | **95.55** | **95.49** | **95.23** |
| 5,000 | 96.69 | 96.75 | 96.79 | 96.79 | 96.70 | 96.66 | 96.61 | 96.58 | 96.47 | 96.60 | 96.54 | 96.37 |
| 6,000 | 97.34 | 97.39 | 97.43 | 97.43 | 97.34 | 97.32 | 97.31 | 97.28 | 97.17 | 97.26 | 97.21 | 97.12 |
| 7,000 | 97.77 | 97.82 | 97.86 | 97.89 | 97.81 | 97.80 | 97.77 | 97.75 | 97.66 | 97.75 | 97.69 | 97.60 |
| 8,000 | **98.20** | **98.25** | **98.26** | **98.30** | **98.23** | **98.24** | **98.22** | **98.17** | **98.14** | **98.20** | **98.11** | **98.07** |
| 9,000 | 98.45 | 98.50 | 98.52 | 98.58 | 98.54 | 98.57 | 98.55 | 98.47 | 98.44 | 98.47 | 98.37 | 98.34 |
| 10,000 – 25,000 | 99.41 | 99.41 | 99.42 | 99.48 | 99.41 | 99.45 | 99.42 | 99.40 | 99.42 | 99.42 | 99.46 | 99.40 |
| Not in the lists | 100 | 100 | 100 | 100 | 100 | 100 | 100 | 100 | 100 | 100 | 100 | 100 |
| Hong Kong | | | | | | | | | | | | |
| Word list | 2010 | 2011 | 2012 | 2013 | 2014 | 2015 | 2016 | 2017 | 2018 | 2019 | 2020 | 2021 |
| 1,000 | 76.54 | 77.03 | 76.21 | 75.69 | 75.20 | 74.38 | 73.94 | 73.88 | 74.51 | 74.11 | 74.72 | 74.92 |
| 2,000 | 87.77 | 88.08 | 87.45 | 87.04 | 86.70 | 86.34 | 86.18 | 86.10 | 86.21 | 85.56 | 85.82 | 85.74 |
| 3,000 | **95.08** | **95.07** | 94.82 | 94.71 | 94.84 | 94.72 | 94.50 | 94.48 | 94.17 | 93.47 | 93.83 | 93.34 |
| 4,000 | 96.69 | 96.71 | **96.59** | **96.58** | **96.61** | **96.53** | **96.40** | **96.39** | **96.11** | **95.65** | **95.69** | **95.28** |
| 5,000 | 97.46 | 97.55 | 97.44 | 97.43 | 97.44 | 97.39 | 97.35 | 97.33 | 97.13 | 96.74 | 96.81 | 96.58 |
| 6,000 | **98.01** | **98.10** | **98.00** | **98.02** | **98.03** | **98.01** | **97.99** | **98.02** | 97.83 | 97.43 | 97.52 | 97.36 |
| 7,000 | 98.34 | 98.47 | 98.38 | 98.38 | 98.37 | 98.37 | 98.37 | 98.40 | **98.26** | **97.95** | **98.00** | 97.83 |
| 8,000 | 98.86 | 98.93 | 98.86 | 98.82 | 98.84 | 98.82 | 98.77 | 98.78 | 98.70 | 98.53 | 98.42 | **98.24** |
| 9,000 | 99.05 | 99.13 | 99.03 | 99.02 | 99.01 | 98.99 | 98.96 | 98.96 | 98.90 | 98.74 | 98.66 | 98.53 |
| 10,000 – 25,000 | 99.70 | 99.76 | 99.73 | 99.69 | 99.67 | 99.66 | 99.66 | 99.68 | 99.68 | 99.65 | 99.70 | 99.66 |
| Not in the lists | 100 | 100 | 100 | 100 | 100 | 100 | 100 | 100 | 100 | 100 | 100 | 100 |
| Ghana | | | | | | | | | | | | |
| Word list | 2010 | 2011 | 2012 | 2013 | 2014 | 2015 | 2016 | 2017 | 2018 | 2019 | 2020 | 2021 |
| 1,000 | 76.33 | 76.84 | 76.54 | 75.75 | 76.24 | 75.81 | 76.89 | 77.28 | 78.45 | 78.01 | 77.33 | 77.24 |
| 2,000 | 87.15 | 87.41 | 87.43 | 86.98 | 87.21 | 87.00 | 87.74 | 87.87 | 88.58 | 88.31 | 88.12 | 87.90 |
| 3,000 | 94.18 | 94.15 | 94.31 | 94.33 | 94.37 | 94.38 | 94.65 | 94.73 | 94.76 | 94.67 | 94.63 | 94.50 |
| 4,000 | **96.00** | **95.96** | **96.09** | **96.15** | **96.09** | **96.21** | **96.32** | **96.31** | **96.41** | **96.33** | **96.38** | **96.23** |
| 5,000 | 97.02 | 97.00 | 97.05 | 97.13 | 97.04 | 97.13 | 97.19 | 97.16 | 97.20 | 97.17 | 97.24 | 97.14 |
| 6,000 | 97.74 | 97.73 | 97.74 | 97.81 | 97.71 | 97.77 | 97.80 | 97.74 | 97.76 | 97.79 | 97.86 | 97.76 |
| 7,000 | **98.20** | **98.20** | **98.19** | **98.24** | **98.12** | **98.16** | **98.17** | **98.09** | **98.09** | **98.16** | **98.24** | **98.12** |
| 8,000 | 98.57 | 98.57 | 98.53 | 98.58 | 98.51 | 98.54 | 98.58 | 98.56 | 98.58 | 98.57 | 98.56 | 98.50 |
| 9,000 | 98.82 | 98.82 | 98.77 | 98.80 | 98.75 | 98.77 | 98.82 | 98.82 | 98.87 | 98.82 | 98.77 | 98.78 |
| 10,000 – 25,000 | 99.60 | 99.58 | 99.52 | 99.53 | 99.47 | 99.47 | 99.44 | 99.45 | 99.49 | 99.50 | 99.51 | 99.48 |
| Not in the lists | 100 | 100 | 100 | 100 | 100 | 100 | 100 | 100 | 100 | 100 | 100 | 100 |
| Canada | | | | | | | | | | | | |
| Word list | 2010 | 2011 | 2012 | 2013 | 2014 | 2015 | 2016 | 2017 | 2018 | 2019 | 2020 | 2021 |
| 1,000 | 80.41 | 80.15 | 79.78 | 79.02 | 78.35 | 78.74 | 77.21 | 77.65 | 78.31 | 78.47 | 77.51 | 77.30 |
| 2,000 | 89.57 | 89.48 | 89.34 | 88.91 | 88.62 | 88.90 | 88.20 | 88.62 | 88.78 | 88.79 | 88.20 | 88.22 |
| 3,000 | 94.63 | 94.63 | 94.65 | 94.62 | 94.64 | 94.73 | 94.67 | 94.82 | 94.78 | 94.75 | 94.68 | 94.73 |
| 4,000 | **96.33** | **96.31** | **96.35** | **96.35** | **96.39** | **96.43** | **96.43** | **96.55** | **96.48** | **96.45** | **96.41** | **96.42** |
| 5,000 | 97.31 | 97.30 | 97.31 | 97.31 | 97.35 | 97.39 | 97.36 | 97.39 | 97.40 | 97.36 | 97.31 | 97.34 |
| 6,000 | **97.92** | **97.91** | **97.92** | **97.92** | **97.94** | **97.96** | **97.95** | **98.06** | **97.99** | **97.96** | **97.90** | **97.94** |
| 7,000 | 98.34 | 98.32 | 98.35 | 98.35 | 98.36 | 98.37 | 98.35 | 98.43 | 98.41 | 98.39 | **98.31** | 98.43 |
| 8,000 | 98.70 | 98.69 | 98.71 | 98.71 | 98.72 | 98.72 | 98.72 | 98.76 | 98.76 | 98.74 | 98.67 | 98.77 |
| 9,000 | 98.93 | 98.93 | 98.94 | 98.95 | 98.95 | 98.96 | 98.94 | 98.98 | 99.00 | 98.98 | 98.89 | 98.98 |
| 10,000 – 25,000 | 99.74 | 99.74 | 99.74 | 99.74 | 99.72 | 99.72 | 99.68 | 99.67 | 99.70 | 99.70 | 99.68 | 99.68 |
| Not in the lists | 100 | 100 | 100 | 100 | 100 | 100 | 100 | 100 | 100 | 100 | 100 | 100 |
| Bangladesh | | | | | | | | | | | | |
| Word list | 2010 | 2011 | 2012 | 2013 | 2014 | 2015 | 2016 | 2017 | 2018 | 2019 | 2020 | 2021 |
| 1,000 | 75.48 | 76.50 | 76.72 | 75.57 | 75.66 | 75.46 | 75.69 | 75.64 | 75.75 | 75.86 | 75.64 | 74.72 |
| 2,000 | 86.37 | 87.02 | 87.19 | 86.46 | 86.85 | 86.78 | 86.80 | 86.86 | 86.77 | 86.82 | 86.48 | 85.75 |
| 3,000 | 93.26 | 93.48 | 93.49 | 93.44 | 93.93 | 94.10 | 93.84 | 93.93 | 93.86 | 93.77 | 93.70 | 93.16 |
| 4,000 | **95.09** | **95.21** | **95.15** | **95.24** | **95.64** | **95.88** | **95.62** | **95.62** | **95.58** | **95.48** | **95.38** | **94.91** |
| 5,000 | 96.21 | 96.27 | 96.24 | 96.30 | 96.61 | 96.83 | 96.59 | 96.59 | 96.61 | 96.52 | 96.45 | 96.16 |
| 6,000 | 96.86 | 96.88 | 96.82 | 96.96 | 97.24 | 97.44 | 97.27 | 97.27 | 97.26 | 97.19 | 97.16 | 96.89 |
| 7,000 | 97.31 | 97.32 | 97.21 | 97.41 | 97.62 | 97.83 | 97.65 | 97.65 | 97.69 | 97.62 | 97.56 | 97.31 |
| 8,000 | 97.72 | 97.70 | 97.57 | 97.81 | **98.02** | **98.21** | **98.05** | **98.08** | **98.07** | **97.98** | **97.91** | 97.71 |
| 9,000 | **97.96** | **97.93** | 97.79 | **98.04** | 98.23 | 98.41 | 98.28 | 98.29 | 98.29 | 98.24 | **98.15** | **97.98** |
| 10,000 | 98.13 | 98.09 | **97.94** | 98.21 | 98.38 | 98.56 | 98.42 | 98.43 | 98.44 | 98.40 | 98.30 | 98.13 |
| 11,000 | 98.28 | 98.23 | 98.10 | 98.36 | 98.52 | 98.69 | 98.56 | 98.56 | 98.58 | 98.55 | 98.42 | 98.26 |
| 12,000 | 98.39 | 98.33 | 98.19 | 98.44 | 98.58 | 98.77 | 98.63 | 98.63 | 98.65 | 98.62 | 98.62 | 98.45 |
| 13,000 | 98.98 | 98.91 | 98.88 | 99.03 | 99.18 | 99.28 | 99.17 | 99.16 | 99.10 | 99.10 | 99.07 | 99.04 |
| 14,000 | 99.05 | 98.98 | 98.93 | 99.10 | 99.26 | 99.35 | 99.24 | 99.22 | 99.16 | 99.17 | 99.12 | 99.09 |
| 15,000 – 25,000 | 99.31 | 99.23 | 99.18 | 99.34 | 99.46 | 99.53 | 99.44 | 99.41 | 99.36 | 99.39 | 99.53 | 99.43 |
| Not in the lists | 100 | 100 | 100 | 100 | 100 | 100 | 100 | 100 | 100 | 100 | 100 | 100 |
| Australia | | | | | | | | | | | | |
| Word list | 2010 | 2011 | 2012 | 2013 | 2014 | 2015 | 2016 | 2017 | 2018 | 2019 | 2020 | 2021 |
| 1,000 | 80.21 | 79.98 | 79.44 | 78.61 | 78.25 | 78.72 | 78.21 | 78.98 | 78.87 | 78.63 | 78.06 | 77.03 |
| 2,000 | 89.45 | 89.35 | 89.08 | 88.70 | 88.50 | 88.78 | 88.51 | 88.92 | 88.56 | 88.39 | 88.07 | 87.16 |
| 3,000 | 94.49 | 94.63 | 94.61 | 94.68 | 94.77 | 94.77 | 94.71 | 94.65 | 94.17 | 94.13 | 94.13 | 93.50 |
| 4,000 | **96.20** | **96.32** | **96.35** | **96.42** | **96.51** | **96.49** | **96.39** | **96.35** | **95.82** | **95.80** | **95.81** | **95.14** |
| 5,000 | 97.13 | 97.26 | 97.27 | 97.35 | 97.44 | 97.44 | 97.34 | 97.30 | 96.82 | 96.76 | 96.76 | 96.21 |
| 6,000 | 97.78 | 97.89 | 97.89 | **97.96** | **98.05** | **98.05** | **97.96** | **97.95** | 97.62 | 97.58 | 97.58 | 97.22 |
| 7,000 | **98.22** | **98.32** | **98.31** | 98.38 | 98.47 | 98.46 | 98.39 | 98.39 | **98.24** | **98.23** | **98.18** | **98.00** |
| 8,000 | 98.61 | 98.68 | 98.69 | 98.74 | 98.81 | 98.80 | 98.77 | 98.75 | 98.76 | 98.76 | 98.69 | 98.68 |
| 9,000 | 98.89 | 98.94 | 98.94 | 98.99 | 99.05 | 99.04 | 99.00 | 98.99 | 98.99 | 98.98 | 98.90 | 98.90 |
| 10,000 – 25,000 | 99.73 | 99.74 | 99.74 | 99.76 | 99.77 | 99.76 | 99.74 | 99.73 | 99.71 | 99.72 | 99.72 | 99.71 |
| Not in the lists | 100 | 100 | 100 | 100 | 100 | 100 | 100 | 100 | 100 | 100 | 100 | 100 |
